# The Einstein–Podolsky–Rosen Steering and Its Certification

**DOI:** 10.3390/e21040422

**Published:** 2019-04-20

**Authors:** Yi-Zheng Zhen, Xin-Yu Xu, Li Li, Nai-Le Liu, Kai Chen

**Affiliations:** 1 Hefei National Laboratory for Physical Sciences at Microscale and Department of Modern Physics, University of Science and Technology of China, Hefei 230026, China; 2 CAS Center for Excellence and Synergetic Innovation Center in Quantum Information and Quantum Physics, University of Science and Technology of China, Hefei 230026, China; 3 Institute for Quantum Science and Engineering, Southern University of Science and Technology (SUSTech), Shenzhen 518055, China

**Keywords:** EPR steering, quantum correlation, non-locality, entanglement, uncertainty relations

## Abstract

The Einstein–Podolsky–Rosen (EPR) steering is a subtle intermediate correlation between entanglement and Bell nonlocality. It not only theoretically completes the whole picture of non-local effects but also practically inspires novel quantum protocols in specific scenarios. However, a verification of EPR steering is still challenging due to difficulties in bounding unsteerable correlations. In this survey, the basic framework to study the bipartite EPR steering is discussed, and general techniques to certify EPR steering correlations are reviewed.

## 1. Introduction

The Einstein–Podolsky–Rosen (EPR) steering [1] depicts one of the most striking features in quantum mechanics: With local measurements, one can steer or prepare a certain state on a remote physical system without even accessing it [2,3]. This feature challenges one’s intuition in a way that the set of prepared states in the EPR steering fashion cannot be produced by any local operations. Therefore, a genuine nonlocal phenomenon happens in this procedure. Whilst EPR steering requires entanglement as the basic resource to complete the remote state preparation task, the correlation implied by EPR steering is not always enough to violate any Bell inequality. In this sense, EPR steering can be seen as a subtle quantum correlation or quantum resource in between entanglement and nonlocality.

The discussion of EPR steering dated back to the emergence of quantum theory, when Einstein, Podolsky, and Rosen questioned the completeness of quantum theory in their famous 1935’s paper [4]. According to their argument on local realism, quantum theory allows a curious phenomenon: the so-called “spooky action at a distance”. In the next year 1936, Schrödinger firstly introduced the terminology “entanglement” and “steering” to describe such quantum “spooky action”. Debates on whether quantum theory is complete and how to understand quantum entanglement lasted for the following 20 years and were finally concluded by Bohm [5] and Bell [6,7]. The celebrated Bell inequality [8] was provided in 1955 as a practical verification of such “spooky action” or equivalent “non-locality”. Noteworthily, the experimental tests of nonlocality without loopholes due to the real devices have been only carried out in recent years [9,10,11,12].

Strictly speaking, Bell inequalities test nonlocal correlations of general physical theories, not necessarily the quantum theory [8]. This can be understood by that Bell inequalities are functions of general probabilities and are independent of how to realize such probabilities. Thus, it is still a question on how quantum theory realizes such “spooky action” in its own context. As proved by Werner in 1989, entanglement is a necessary resource to exhibit nonlocality but not a sufficient one [13]. Note that, in some physics research fields, e.g., condensed matters, “entanglement” is equivalently used as “nonlocality” to discuss the genuine quantum phenomenon. In Werner’s paper [13], the disentangled state is termed as the “classical correlated state”, when the terminology “separable state” was not often used at that time. It, thus, drives physicists to consider under what conditions can entanglement show nonlocal effects in the quantum context.

This problem was further addressed by Wiseman, Jones, and Doherty [1,14] in 2007. They showed that there exists a set of bipartite entangled states, which can exhibit EPR steering properties but are not sufficient to violate Bell inequalities. For such states, termed as “EPR steerable states”, one party can remotely prepare certain quantum states on the other party, and such preparations can not be replaced by any classical or quantum local operations. It, thus, represents another form of “spooky action at a distance”. This “action” is in the quantum context in the sense that the description on the other party is always quantum. Then, EPR steering stands as an intermediate between entanglement and nonlocality, and they together form a relatively complete picture. On the one hand, EPR steering can be seen as a certification of entanglement. On the other hand, EPR steering exhibits a weaker form of nonlocality in specific scenarios.

The significance of studying EPR steering follows from important applications of entanglement and nonlocality. The entanglement and nonlocality have been proved to be important resources for many quantum information tasks, from quantum communications to quantum computation. As an intermediate but subtle resource, EPR steering may help to reduce the difficulty of such tasks and helps to inspire new protocols. For instance, nonlocality offers the strongest security in quantum cryptography. Nevertheless, the realization of nonlocality is based on violating Bell inequality, which is experimentally difficult. Simultaneously, violating EPR steering inequality is relatively applicable [15,16,17], and the realization of EPR steering also provides a different communication security for specific tasks [18].

Numerous results have been concluded in recent years. To certify EPR steering, there have been many approaches to witness EPR steerable correlations. Besides the basic linear inequality [19,20], local uncertainty relations [21,22,23], entropic uncertainty relations [24], fine-grained uncertainty relations [25], the CHSH-type inequality [26], covariance matrices [27], the semidefinite programming method [28], the all-versus-nothing fashion [29,30], and other methods, have been adopted in formulating inequalities and equations to verify EPR steering. As for understanding EPR steering, the asymmetric property [31,32], the super-activation of EPR steering correlation [33,34], the quantization of EPR steering [34,35,36,37], the negativity of steerable states [38], steering in the presence of positive operator valued measure (POVMs) [39], the resource theory description [40], the multipartite case [41,42], etc. are deeply investigated. In addition, relations between EPR steering and the uncertainty principle [23,24,43,44], joint measurability [45,46], sub-channel discrimination [47], etc. have also been discussed in the literature. Experimentally, EPR steering has been tested on various physical systems and platforms [16,19,48,49,50,51,52,53].

Noteworthily, comprehensive reviews [21,22,28] have given a complete picture of EPR steering. In Reference [21], the EPR steering is introduced based on the EPR *Gedankenexperiment* [4], while proposals to realize EPR steering test are reviewed from both the theoretical and experimental perspectives. The experimental friendly criteria for certifying EPR steering is thoroughly investigated in Reference [22]. In particular, the characterization of EPR steering is reviewed through the semidefinite programming method [28], which can be explicitly used to tackle the complicated numerical problems in detecting EPR steering. Recently, the EPR steering test is further generalized to a unified framework where classical, quantum, and post-quantum steering can be investigated [54]. The black box framework in that paper is the same with the framework adopted here.

In this paper, we will mainly focus on the basic techniques to certify the bipartite EPR steering and related quantum correlations, and show how to certify EPR steerable correlation in different fashions. This survey is organized as follows. In Section 2, the basic notations and the box framework combined with trust/untrust scenarios will be introduced. After a brief discussion of entanglement and nonlocality in such a framework, EPR steering as well as other equivalent descriptions will be introduced in Section 3. In Section 4, the systematic method to formulate the criteria for certifying EPR steering will be discussed. Two types of criteria, (a) linear EPR steering inequality and (b) criterion based on uncertainty relations, will be studied in detail. Their performances on some typical states will also be given. Finally, a summary will be given in Section 5.

## 2. Preliminaries and Notations

In this paper, we will focus on the bipartite correlation $P\left(ab|xy\right)$ with input parameters x,y and output parameters a,b and discuss, under certain assumptions, whether the correlation can be certified as EPR steerable. Before the discussion, we firstly introduce the basic terminology and the notations that will be used throughout the paper.

### 2.1. The Box Framework

**Figure 1:**
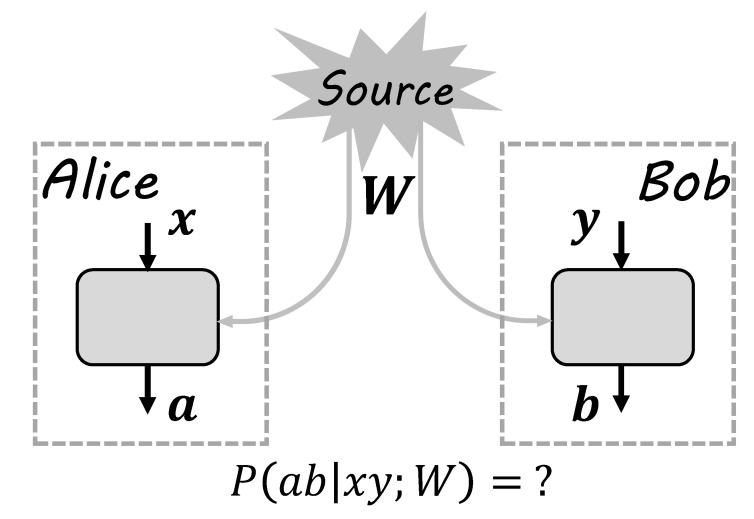
The box framework: The source distributes state *W* to Alice and Bob. In their own closed labs, Alice and Bob make operations on received local states. Alice’s operations are labeled by inputs *x*, with outputs labeled by *a*. Bob’s operations are labeled by inputs *y*, with outputs labeled by *b*. After the experiment, Alice and Bob publicize their results and the corresponding statistics are denoted by probability distribution $\left\{P\left(ab|xy;W\right)\right\}$. According to such a distribution, the local property of *W* can be inferred.

A typical experiment of testing a bipartite correlation can be described by the box framework, as shown in Figure 1. Suppose two parties, Alice and Bob, are in their closed labs to do the experiment. The lab is sketched as the doted rectangle, inside which there is an experimental device sketched as the solid rectangle. In each run of the experiment, Alice and Bob are distributed with a bipartite state *W* from a source, which may be unknown. In their own labs, combined with the subsystem they received, Alice and Bob can input *x* and *y* to the device and obtain outputs *a* and *b*, respectively. Such a run is repeated enough times so that, after the experiment, Alice and Bob can obtain the correlation $P\left(ab|xy\right)$ by announcing their input and output results. Depending on different descriptions and mechanics of the source and device, the correlation may have different structures and properties. The aim of the box framework is then to characterize the dependence of the correlation on descriptions of sources and devices.

In general, there is no restrictions on the source, inputs, and outputs. For instance, the source *W*; inputs x,y; and outputs a,b can all be quantum states, with the devices being quantum instruments. In this case, the box framework characterizes general local quantum operations on bipartite quantum states. In this paper, we will restrict the device to be the typical measurement device in labs. That is, the inputs x,y represent different measurement settings on the received subsystem and the outputs a,b represent different outcomes. Physically, x,y,a,b can be described by natural numbers 0,1,2,⋯ and corresponding sets are denoted as X,Y,A,B, respectively. In the scenario of steering and nonlocality, there are some common assumptions.

#### 2.1.1. The No-Signaling Principle

Roughly speaking, the no-signaling principle describes that Alice and Bob cannot communicate with each other during the test [55,56]. In the above box framework, this principle guarantees the independence between Alice and Bob such that the correlation $P\left(ab|xy\right)$ is faithfully generated by the state *W* and measurements but not any other statistics shared before or during the test. Mathematically, the no-signaling principle has the following form,

(1) $$\begin{array}{cc} \sum_{a}P\left(ab|xy;W\right) & =P\left(b|xy;W\right)=P\left(b|y;W\right),\forall x\in X, \end{array}$$

(2) $$\begin{array}{cc} \sum_{b}P\left(ab|xy;W\right) & =P\left(a|xy;W\right)=P\left(a|x;W\right),\forall y\in Y. \end{array}$$

Therefore, the no-signaling principle denies the possibility that Alice and Bob can guess each other’s measurement setting *y* or *x* based on their local statistics $P\left(a|x;W\right)$ or $P\left(b|y;W\right)$, respectively.

Experimentally, this principle is guaranteed by Alice and Bob being separated far away (space-like separation) and by both of them choosing measurement settings independently and randomly. The no-signaling principle is then guaranteed by two hypotheses. Firstly, two parties in the space-like separation cannot communicate with each other. Secondly, the random number generators [57] in Alice’s and Bob’s labs should be truly independent and random.

In the test of nonlocality and EPR steering, we suppose that the no-signaling principle has been guaranteed.

#### 2.1.2. Trust and Untrust

If the description of boxes is restricted as quantum or classical, we can further define if a device is trusted or not for the sake of practice. A device is said to be trusted if it is believed that the function of the device is exactly what we expect. This definition comes from the sense that, without the assistance of other resources, it is, in principle, impossible to verify how an unknown device really functions based solely on statistics of measurement results. Particularly, in the rest of the paper, the device is trusted if it is a quantum device and the accurate quantum mechanical description is known.

Therefore, if we say some devices are trusted, we actually make additional assumptions. For instance, we say a measurement device is trusted if its measurement can be exactly described by a known set of POVMs $\left\{E_{b}^{y}\right\}$, where *y* is the measurement settings and *b* is the measurement outcome. On the contrary, we say a measurement device is untrusted if we can, at most, describe the measurement results by a probability distribution $P\left(b|y\right)$.

The scenario is device-independent if all devices and the source are untrusted. Particularly, the scenario is measurement-device-independent if all measurement devices are untrusted. If some but not all measurement devices are untrusted, we say the corresponding scenario as semi-measurement-device-independent.

### 2.2. Entanglement and Nonlocality

In the box framework, we can discuss entanglement and nonlocality in an operational manner. Let λ label different hidden states in *W* and $p_{\lambda }$ be its probability such that $\int d\lambda p_{\lambda }=1$. The correlation can be written as

(3) $$P\left(ab|xy;W\right)=\int d\lambda p_{\lambda }P\left(ab|xy,\lambda \right).$$

The local realism argues that, for any hidden variable λ, $P\left(ab|xy,\lambda \right)$ can be localized such that $P\left(ab|xy,\lambda \right)=P\left(a|x,\lambda \right)P\left(b|y,\lambda \right)$. We say the correlation $P\left(ab|xy;W\right)$ is a local correlation if all hidden states in Equation (3) can be localized.

The nonlocality is defined as the failure of local realism, usually modeled by local hidden variable (LHV) models. The main property of LHV models is that, if two parties are no longer interacting (guaranteed by space-like separation), their measurements should be local, i.e., *a* should be independent on *y* and *b* (similarly for *b*). Thus, for each hidden variable λ, the LHV models produce a localized correlation $P\left(ab|xy,\lambda \right)=P\left(a|x,\lambda \right)P\left(b|y,\lambda \right)$. The nonlocal correlation is defined as correlations that cannot explained by the local correlation

(4) $$P_{LHV}\left(ab|xy;W\right)=\int d\lambda p_{\lambda }P\left(a|x,\lambda \right)P\left(b|y,\lambda \right),$$

where $P\left(a|x,\lambda \right)$ and $P\left(b|y,\lambda \right)$ are arbitrary probabilities. If the statistic of the experimental results cannot be explained by Equation (4), then the correlation is nonlocal and we say the source *W* is nonlocal.

The Bell inequality is indeed a linear constraint on all local correlations. This is based on the fact that all local correlations from Equation (4) form a convex subset. There are some correlations produced by quantum mechanics outside this subset. Precisely, in the probability space, points of local correlations form a polytope, while all probabilities produced by quantum mechanics form a superset of the polytope [8]. Thus, one can distinguish a specific nonlocal correlation from all local correlations by a linear equation. Additionally, since Alice’s and Bob’s measurement results are described by general probabilities, the problem of nonlocality corresponds to the device-independent scenario.

The entanglement is defined as the failure of description in the form of separable states. The separable states have a clear definition that $\rho_{SEP}$ is separable if $\rho_{SEP}=\sum_{k}p_{k}\rho_{k}^{A}⊗\rho_{k}^{B}$ with $\rho_{k}^{A}$ and $\rho_{k}^{B}$ being some local quantum states and $\sum_{k}p_{k}=1$. Usually, the decomposition of a separable state is not unified and the verification of a separable is not a easy task. However, if the source *W* distributes separable states in the box framework, then the correlation is in the form of

(5) $$P_{SEP}\left(ab|xy;W\right)=\int d\lambda p_{\lambda }P_{Q}\left(a|x,\lambda \right)P_{Q}\left(b|y,\lambda \right),$$

where $P_{Q}\left(a|x,\lambda \right)=\mathrm{tr}\left[E_{a}^{x}\rho_{\lambda }^{A}\right]$ and $P_{Q}\left(b|y,\lambda \right)=\mathrm{tr}\left[F_{b}^{y}\rho_{\lambda }^{B}\right]$ are probabilities yielded by quantum measurements. Here $\rho_{\lambda }^{A}$ and $\rho_{\lambda }^{B}$ are local hidden quantum states which may be unknown to Alice and Bob, while $E_{a}^{x}$ and $F_{b}^{y}$ are POVMs that Alice and Bob know well. If the statistic of experimental results cannot be explained by Equation (5), then the correlation is non-separable, i.e., entangled, and we say the source *W* is entangled.

Like the Bell inequality, one can use a linear constraint, the so-called entanglement witness, to bound all separable correlations to certify an entangled correlation. Similar to the case of local correlations, correlations produced by all separable states also form a convex set. Since all devices are assumed to be quantum, here, the entanglement corresponds to the scenario where all measurement devices are trusted.

## 3. The EPR Steering

### 3.1. Definition

From the above introduction, it is easy to see that definitions of nonlocality and entanglement have two similarities. Firstly, both of them are defined by the failure of corresponding local models in their own contexts, i.e., LHV models and separable quantum states, respectively. Secondly, as for the two local models, the descriptions on Alice’s and Bob’s systems are symmetric, i.e., general probabilities $P\left(a|x,\lambda \right)$ and $P\left(b|y,\lambda \right)$ in LHV models and quantum probabilities $P_{Q}\left(a|x,\lambda \right)$ and $P_{Q}\left(b|y,\lambda \right)$ in separable states. The only difference between the two definitions is whether the local descriptions are both quantum. A natural equation would be “What if the local descriptions are asymmetric?” and “Can this asymmetric property lead to novel correlations?”. The answer is yes. The corresponding local model is called the local hidden state (LHS) model and its failure implies the main objective of this paper, the correlation of EPR steering [1].

**Definition** **1** (EPR steering). *In a box frame test, the experimental result statistics exhibits EPR steering property, if it cannot be explained by the correlation of LHS models, i.e., the correlation cannot be written as*

(6) $$P_{LHS}\left(ab|xy;W\right)=\int d\lambda p_{\lambda }P\left(a|x,\lambda \right)P_{Q}\left(b|y,\lambda \right),$$

*where $p_{\lambda }$ is a probability distribution satisfying $\int d\lambda p_{\lambda }=1$, $P\left(a|x,\lambda \right)$ is an arbitrary probability distribution, and $P_{Q}\left(b|y,\lambda \right)=\mathrm{tr}\left[F_{b}^{y}\sigma_{\lambda }\right]$ is a probability distribution generated by POVM $F_{b}^{y}$ on quantum state $\sigma_{\lambda }$.*

*It is said that the corresponding quantum state is EPR steerable if Equation (6) is violated.*

**Figure 2:**
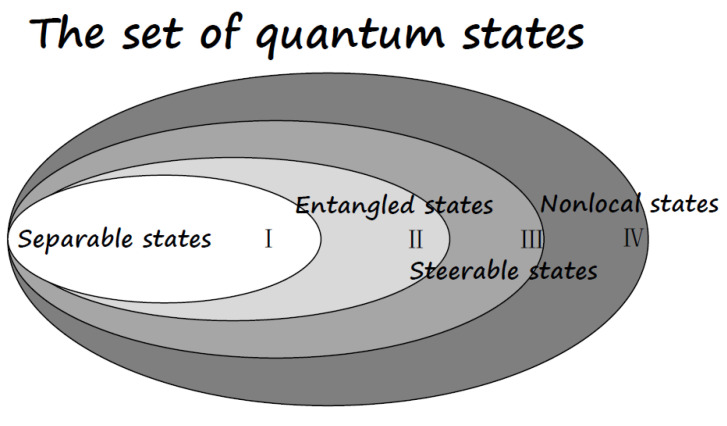
The set of quantum states: All quantum states form a convex set, with the boundary being the pure state. The region I represents the convex subset of separable states. The complement set, i.e., regions II, III, and IV, represent entangled states. Particularly, regions III and IV represent Einstein–Podolsky–Rosen (EPR) steerable states, and the region IV represents nonlocal states. Region II are entangled states which is neither EPR steerable nor nonlocal.

The relationship among EPR steerable states, entangled states, and nonlocal states are sketched out in Figure 2.

### 3.2. One-Sided Measurement Device Independence

The understanding of EPR steering can be more clear if we discuss it in the trust and untrust scenarios. As has been discussed before, nonlocality defies a local correlation in the device-independent scenario, while entanglement defies local correlations in the measurement-dependent scenario. Since EPR steering is defined as the failure of LHS models, where only one party is assumed to be quantum, we have the following claim.

**Remark** **1.** *EPR steering defies all local correlations in the one-sided measurement-device-independent scenario.*

*This scenario corresponds to the real situation when users in the communication task need different levels of security. For instance, in the communication task between banks and individuals, obviously it is easier for banks to prepare their devices to be trustworthy. For individuals, however, due to limits of costs and environments, their devices are hard to be guaranteed as trustworthy ones. In this case, let individuals be Alice and banks be Bob, such that if EPR steering correlation is certified by the violation steering inequality, then the secure quantum communications can be achieved [18].*

**Figure 3:**
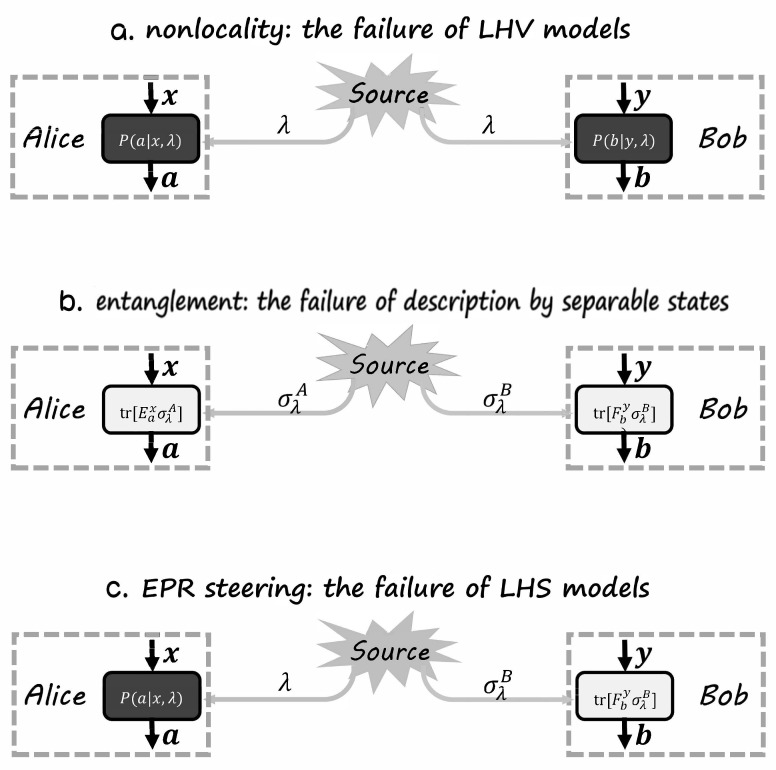
The box framework for nonlocality, entanglement, and EPR steering. The color black represents untrusted, gray represents unknown, and white represents trusted. (**a**) The nonlocality scenario, where the source is unknown and measurement devices are untrusted. (**b**) The entanglement scenario, where source is unknown and measurement devices are trusted. (**c**) The EPR steering scenario, where source is unknown and Alice’s measurement devices are untrusted while Bob’s are trusted.

Different scenarios corresponding to nonlocality, entanglement, and EPR steering are shown in Figure 3.

### 3.3. Schrödinger’s Steering Theorem

As an equivalent definition, one can consider the assemblage. The assemblage is defined as the collection of ensembles, denoted by ${\left\{{\tilde{\rho }}_{a|x}\right\}}_{a,x}$, where ${\tilde{\rho }}_{a|x}$ are unnormalized quantum states satisfying $\sum_{a}{\tilde{\rho }}_{a|x}=\sigma ,$ ∀x. The definition of EPR steering can be applied on the assemblage ${\left\{{\tilde{\rho }}_{a|x}\right\}}_{a,x}$ instead of correlations P(ab|xy). This equivalence is guaranteed by the Schrödinger’s steering theorem [2,3].

**Theorem** **1** (Schroödinger’s steering theorem). *The following two statements hold:*

*1.*  *For any quantum state $\rho_{AB,}$, let ${\left\{E_{a}^{x}\right\}}_{a}$ be a complete set of POVMs satisfying $\sum_{a}E_{a}^{x}=I$, ∀x. Then, the conditional states ${\tilde{\rho }}_{B}^{a|x}={\mathrm{tr}}_{A}\left[E_{a}^{x}⊗I\rho_{AB}\right]$ for all x and a form an assemblage.*
*2.*  *For any assemblage ${\left\{{\tilde{\rho }}_{a|x}\right\}}_{a,x}$ with $\sum_{a}{\tilde{\rho }}_{a|x}=\sigma$, there always exist a pure quantum state ${|\psi \rangle }_{AB}$ satisfying ${\mathrm{tr}}_{A}\left[{|\psi \rangle }_{AB}\langle \psi |\right]=\sigma$ and complete sets of POVMs $\left\{E_{a}^{x}\right\}$ satisfying $\sum_{a}E_{a}^{x}=I$ for all x, such that ${\tilde{\rho }}_{a|x}$ can be produced, i.e., ${\tilde{\rho }}_{a|x}={\mathrm{tr}}_{A}\left[E_{a}^{x}⊗I{|\psi \rangle }_{AB}\langle \psi |\right]$.*

**Proof.**  For the first statement, it is straightforward to verity that, for all *x*,

$$\sum_{a}{\tilde{\rho }}_{B}^{a|x}=\sum_{a}{\mathrm{tr}}_{A}\left[E_{a}^{x}⊗I\rho_{AB}\right]={\mathrm{tr}}_{A}\left[\sum_{a}E_{a}^{x}⊗I\rho_{AB}\right]={\mathrm{tr}}_{A}\left[\rho_{AB}\right]=\rho_{B}.$$

For the second statement, write $\rho_{B}$ in its diagonal form $\rho_{B}=\sum_{i}\lambda_{i}|i\rangle \langle i|$ with $\lambda_{i}>0$ and let *D* be the diagonal matrix $D=\mathrm{diag}\left[\sqrt{\lambda_{1}},\ldots ,\sqrt{\lambda_{d}}\right]$. Denote the generalized invertible matrix of *D* as $D^{-1}$. It can be verified that $E_{a}^{x}=D^{-1}{\tilde{\rho }}_{a|x}^{T}D^{-1}$ and ${|\psi \rangle }_{AB}=\sum_{i}\sqrt{\lambda_{i}}|ii\rangle$ are required POVMs and the quantum state, respectively. □

Then, an assemblage ${\left\{\rho_{a|x}\right\}}_{a,x}$ is said to be unsteerable if it can be produced by rearrangement on an LHS model $\left\{p_{\lambda }\sigma_{\lambda }\right\}$, i.e., $\rho_{a|x}=\sum_{\lambda }p_{x,\lambda }\left(a\right)p_{\lambda }\sigma_{\lambda }$ with $\sum_{a}p_{x,\lambda }\left(a\right)=1$ for all *x* and λ. Particularly, for two-qubit states, the steered states ${\tilde{\rho }}_{B}^{a|x}$ form an ellipsoid in the Bloch sphere on Bob’s side [58]. The volume of such ellipsoid indicates the steerability of the bipartite state. If the assemblage cannot be written in this manner, it is said to be EPR steerable. This EPR steering definition is equivalent to Definition 1 on the condition that Bob is allowed to do the state tomography for each conditional state $\rho_{a|x}$. Furthermore, in Reference [54] post-quantum steering is well-studied using no-signaling assemblages. If Bob’s measurements are not sufficient to do the tomography, then it is hard for him to obtain each $\rho_{a|x}$ yet to verify the EPR steerability. In this case, however, the statistic of measurement results $P\left(ab|xy\right)$ is still useful. In the following discussion, Definition 1 will be mainly considered.

There is an interesting analog of the assemblage [59], from the perspective of the state-channel duality [60]. If the set of local hidden state $\sigma_{\lambda }$ is replaced with a set of POVMs $\left\{G_{\lambda }\right\}$, then the assemblage of $\left\{G_{\lambda }\right\}$ can be defined as jointly measurable observables. That is, a set of POVMs $\left\{E_{a}^{x}\right\}$ is jointly measurable if $E_{a}^{x}=\sum_{\lambda }p_{x,\lambda }\left(a\right)p_{\lambda }G_{\lambda }$, with $p_{x,\lambda }\left(a\right)$ being probabilities. It has been proved that a given assemblage ${\left\{\rho_{a|x}\right\}}_{a,x}$ is unsteerable if and only if Alice’s measurements ${\left\{E_{a}^{x}\right\}}_{a,x}$ is jointly measurable [45,46], which can be checked from the Proof of Theorem 1.

## 4. Criteria of EPR Steering

A natural question arises on how to certify the EPR steering correlation. It can be shown that unsteerable correlations, i.e., correlations produced by LHS models, form a convex subset. According to the hyperplane separate theorem, there always exists a linear constraint of all unsteerable correlations, such that steerable ones can be witnessed [22].

Suppose that the box framework is fixed, i.e., X, Y, A, and B are all fixed. Then, the set of probability distributions $\left\{P\left(ab|xy\right)|x\in X,y\in Y,a\in A,b\in B\right\}$ can be seen as a point in the probability space. All correlations yielded by LHS models in Equation (6) $\left\{p_{\lambda }\sigma_{\lambda }\right\}$ form a subset $\left\{P_{LHS}\left(ab|xy\right)|x\in X,y\in Y,a\in A,b\in B\right\}$. This subset of usteerable correlations is convex.

**Lemma** **1.** *The unsteerable correlations $\left\{P_{LHS}\left(ab|xy\right)\right\}$ form a convex subset.*

**Proof.**  For any two LHS models $\left\{p_{\lambda }^{\left(1\right)}\sigma_{\lambda }^{\left(1\right)}\right\}$ and $\left\{p_{\mu }^{\left(2\right)}\sigma_{\mu }^{\left(2\right)}\right\}$, the correlation yielded by them are

(7) $$\begin{array}{cc} P_{LHS}^{\left(1\right)}\left(ab|xy\right) & =\int dp_{\lambda }^{\left(1\right)}P^{\left(1\right)}\left(a|x,\lambda \right)\mathrm{tr}\left[F_{b}^{y}\sigma_{\lambda }^{\left(1\right)}\right], \end{array}$$

(8) $$\begin{array}{cc} P_{LHS}^{\left(2\right)}\left(ab|xy\right) & =\int dp_{\mu }^{\left(2\right)}P^{\left(2\right)}\left(a|x,\mu \right)\mathrm{tr}\left[F_{b}^{y}\sigma_{\mu }^{\left(2\right)}\right], \end{array}$$

respectively. Then, any linear combination of these two, i.e., $tP_{LHS}^{\left(1\right)}\left(ab|xy\right)+\left(1-t\right)P_{LHS}^{\left(2\right)}\left(ab|xy\right)$ with 0⩽t⩽1, can always be written as the correlation yielded by another LHS model $\left\{q_{\nu }\tau_{\nu }\right\}$, where

(9) $$\begin{array}{cc} q_{\nu } & =tp_{\lambda }^{\left(1\right)}\delta_{\nu \lambda }+\left(1-t\right)p_{\mu }^{\left(2\right)}\delta_{\nu \mu }, \end{array}$$

(10) $$\begin{array}{cc} \tau_{\nu } & =\sigma_{\lambda }^{\left(1\right)}\delta_{\nu \lambda }+\sigma_{\mu }^{\left(2\right)}\delta_{\nu \mu }. \end{array}$$

It is easy to verify that

(11) $$\begin{array}{cc} P_{LHS}^{\left(3\right)}\left(ab|xy\right)= & \int dq_{\nu }P\left(a|x,\nu \right)\mathrm{tr}\left[F_{b}^{y}\tau_{\nu }\right] \end{array}$$

(12) $$\begin{array}{cc} = & t\int dp_{\lambda }^{\left(1\right)}P^{\left(1\right)}\left(a|x,\lambda \right)\mathrm{tr}\left[F_{b}^{y}\sigma_{\lambda }^{\left(1\right)}\right] \end{array}$$

(13) $$\begin{array}{c} +\left(1-t\right)\int dp_{\mu }^{\left(2\right)}P^{\left(2\right)}\left(a|x,\mu \right)\mathrm{tr}\left[F_{b}^{y}\sigma_{\mu }^{\left(2\right)}\right] \end{array}$$

(14) $$\begin{array}{cc} = & tP_{LHS}^{\left(1\right)}\left(ab|xy\right)+\left(1-t\right)P_{LHS}^{\left(2\right)}\left(ab|xy\right). \end{array}$$

Therefore, the subset of all unsteerable correlation is convex. □

Any convex subset can be bounded by a linear equation, which is guaranteed by the hyperplane separation theorem [61].

**Lemma** **2.** *(Hyperplane separation theorem) Let A and B be two disjoint nonempty convex subsets of $R_{n}$. Then, there exists a nonzero vector v and a real number c such that*

〈x,v〉≥cand〈y,v〉≤c

*for all x in A and y in B, i.e., the hyperplane 〈·,v〉=c and v the normal vector, separates A and B.*

The proof can be found in many Linear Algebra textbooks (like Reference [61]) and is skipped here. Based on these two lemmas, one can certify EPR correlations by linear inequalities [22].

**Theorem** **2.** *Any EPR steerable correlation can be verified by an inequality.*

**Proof.**  According to Lemma 2, let the set *A* be the set of all unsteerable correlations, which is proved by Lemma 1. For any EPR steerable correlation $P_{STE}\left(ab|xy\right)$, let *B* be a sufficient open ball containing $P_{STE}\left(ab|xy\right)$, such that the open ball is disjoint with the subset *A*. Then, there always exists a hyperplane $v\left(P\left(ab|xy\right)\right)=\sum_{abxy}v_{ab}^{xy}P\left(ab|xy\right)=c$, such that $v\left(P_{LHS}\left(ab|xy\right)\right)⩾c$ holds for all unsteerable correlations $P_{LHS}\left(ab|xy\right)$ while $v\left(P_{STE}\left(ab|xy\right)\right)<c$ holds for the certain EPR steerable correlation $P_{STE}\left(ab|xy\right)$. □

### 4.1. Linear EPR Steering Inequality

Perhaps the most straightforward criteria to verify EPR steering is the linear steering inequality. The linear steering inequality to certify EPR steering is like the Bell inequality to nonlocality and the entanglement witness to entanglement. From the Proof of Theorem 2, the linear steering inequality has a general from, i.e., for all unsteerable correlations, the following inequality holds:

(15) $$\begin{array}{cc} I\left(P\right) & =\sum_{a,b,x,y}V_{ab}^{xy}P\left(ab|xy\right)⩽B_{LHS}, \end{array}$$

(16) $$\begin{array}{cc} B_{LHS} & =\max_{P_{LHS}}\sum_{a,b,x,y}V_{ab}^{xy}P_{LHS}\left(ab|xy\right), \end{array}$$

where $P=\left\{P\left(ab|xy\right)\right\}$ denotes the correlation $\left\{P\left(ab|xy\right)|a\in A,b\in B,x\in X,y\in Y\right\}$, $V_{ab}^{xy}\in R$ are some coefficients, and $B_{LHS}$ is the bound of all unsteerable correlations.

Then, if for a certain correlation $Q=\left\{Q\left(ab|xy\right)\right\}$ satisfies $I\left(Q\right)>B_{LHS}$, i.e., the linear steering inequality in Equation (15) is violated, then it can be conclude that Q cannot be explained by any LHS correlations, i.e., Q is EPR steerable.

In practice, the expectation value of the measurement results is usually considered for convenience and clarity. Combined with the scenario of EPR steering where Alice’s and Bob’s measurement devices are untrusted and trusted, respectively, denote $A_{x}=\left\{a_{x}\in R\right\}$ as the random variable corresponding to Alice’s measurements and $B_{y}=\sum_{b}b_{y}F_{b}^{y}$ as the general quantum measurement for Bob’s measurements, with $F_{b}^{y}$ being the POVM corresponding to the result $b_{y}$.

Suppose that, in an EPR steering test experiment, Alice and Bob randomly and independently choose *n* pairs of measurements $A_{k}$ and $B_{k}$, respectively, labeled by k=1,2,⋯,n. After the experiment, the value of each pair of measurements is

(17) $$\langle A_{k}B_{k}\rangle =\sum_{a_{k},b_{k}}a_{k}b_{k}P\left(ab|A_{k}B_{k}\right).$$

Then, the following linear steering inequality holds for all unsteerable correlations [19,20].

**Theorem** **3** (The linear EPR steering inequality). *If the result of an EPR steering test violates the following inequality*

(18) $$S_{n}=\frac{1}{n}\sum_{k=1}^{n}g_{k}\langle A_{k}B_{k}\rangle ⩽C_{n},$$

*where $g_{k}$ are real numbers and $C_{n}$ satisfies*

(19) $$C_{n}=\max_{a_{k}\in A_{k}}\left\{\lambda_{max}\left(\frac{1}{n}\sum_{n=1}^{n}g_{k}a_{k}B_{k}\right)\right\},$$

*with $\lambda_{\max}\left(\cdot \right)$ the maximal eigenvalue of the matrix, then the correlation of the test shows EPR steering. The corresponding quantum state $\rho_{AB}$ is EPR steerable, and more precisely, Alice can steer Bob.*

**Proof.**  By definition, $S_{n}⩽C_{n}$ is an EPR steering inequality when it holds for all unsteerable correlation $P_{LHS}$. $P_{LHS}$ has a general form as defined by Equation (6), i.e.,

$$P_{LHS}\left(ab|xy\right)=\int dp_{\lambda }P\left(a|x,\lambda \right)\mathrm{tr}\left[F_{b}^{y}\sigma_{\lambda }\right].$$

It is straightforward to verify that

(20) $$\begin{array}{cc} S_{n}\left(P_{LHS}\right) & =\frac{1}{n}\sum_{k=1}^{n}g_{k}\sum_{a_{k}}a_{k}\int dp_{\lambda }P\left(a_{k}|A_{k},\lambda \right)\mathrm{tr}\left[B_{k}\sigma_{\lambda }\right] \end{array}$$

(21) $$\begin{array}{c} ⩽\max_{a_{k}\in A_{k}}\frac{1}{n}\sum_{k=1}^{n}g_{k}a_{k}\int dp_{\lambda }\mathrm{tr}\left[B_{k}\sigma_{\lambda }\right] \end{array}$$

(22) $$\begin{array}{c} ⩽\max_{a_{k}\in A_{k}}\lambda_{\max}\left(\frac{1}{n}\sum_{k=1}^{n}g_{k}a_{k}B_{k}\right) \end{array}$$

(23) $$\begin{array}{c} =C_{n}. \end{array}$$

Here, the second line comes from $\sum_{a_{k}}a_{k}P\left(a_{k}|A_{k},\lambda \right)\mathrm{tr}\left[B_{k}\sigma_{\lambda }\right]\leq \max_{a_{k}\in A_{k}}a_{k}\mathrm{tr}\left[B_{k}\sigma_{\lambda }\right]$, and the third line comes from

(24) $$\begin{array}{cc} \frac{1}{n}\sum_{k=1}^{n}g_{k}a_{k}\int dp_{\lambda }\mathrm{tr}\left[B_{k}\sigma_{\lambda }\right] & =\mathrm{tr}\left[\left(\frac{1}{n}\sum_{k=1}^{n}g_{k}a_{k}B_{k}\right)\int dp_{\lambda }\sigma_{\lambda }\right]⩽\lambda_{max}\left(\frac{1}{n}\sum_{k=1}^{n}g_{k}a_{k}B_{k}\right). \end{array}$$

 □

Here, $g_{k}$ are flexible coefficients to help to form efficient inequalities.

**Example** **1.** *The 2-qubit Werner state [13].*

*As a simple example, one can consider the 2-qubit Werner state, which is an often-used bipartite quantum states in quantum information processes. It can be constructed as the mixture of the maximally entangled state $|\Psi^{-}\rangle =\left(|01\rangle -|10\rangle \right)/\sqrt{2}$ and the white noise I/4, i.e.,*

(25) $$W_{\mu }=\mu |\Psi^{-}\rangle \langle \Psi^{-}|+\left(1-\mu \right)\frac{I}{4},$$

*where $\mu \in \left[0,1\right]$. It can be theoretically proved that $W_{\mu }$ is entangled when μ>1/3 and is separable when μ≤1/3 [13]. When $\mu >1/\sqrt{2}$, there exists certain observables such that the CHSH inequality is violated [62], i.e., $W_{\mu }$ is nonlocal when $\mu >1/\sqrt{2}$. When μ≲0.66, any measurement results of $W_{\mu }$ can be explained by some LHV models, i.e., $W_{\mu }$ never exhibits a nonlocality when μ≲0.66 [63]. It is an open question of whether $W_{\mu }$ is nonlocal when $0.66≲\mu \leq 1/\sqrt{2}$.*

*It has been proved that $\mu >\frac{1}{2}$ is the critical bound for the EPR steerability of $W_{\mu }$ [1], i.e., any measurement results of $W_{\mu }$ can be explained by LHS models when $\mu \leq \frac{1}{2}$.*

*It is easy to see that the performance of linear EPR steering inequality (Theorem 3) depends on the number of Alice and Bob’s measurement pairs and Bob’s observables. Furthermore, from the symmetric property of the 2-qubit Werner state, when Bob’s k’th observable is $B_{k}=n_{k}\cdot \sigma$, where $n_{k}=\left(n_{x}^{\left(k\right)},n_{y}^{\left(k\right)},n_{z}^{\left(k\right)}\right)$ is a unit vector and $\sigma =\left(\sigma_{x},\sigma_{y},\sigma_{z}\right)$ is the set of Pauli matrices, i.e.,*

(26) $$\sigma_{x}=\left(\begin{array}{cc} 0 & 1\\ 1 & 0 \end{array}\right),\sigma_{y}=\left(\begin{array}{cc} 0 & -i\\ i & 0 \end{array}\right),\sigma_{z}=\left(\begin{array}{cc} 1 & 0\\ 0 & -1 \end{array}\right),$$

*Alice can always choose her observable as $A_{k}=-n_{k}\cdot \sigma$, such that the expectation value of the measurement pair $\mathrm{tr}\left[A_{k}⊗B_{k}W_{\mu }\right]=\mathrm{tr}\left[-n_{k}\cdot \sigma ⊗n_{k}\cdot \sigma W_{\mu }\right]=\mu$. If we further let $g_{k}=1$, $S_{n}=\mu$ always holds independent of the number of measurements.*

*The bound $C_{n}$, however, depends on n and the form of $B_{k}$. More precisely, when n=2, let $B_{1}=\sigma_{x}$ and $B_{2}=\sigma_{y}$. The corresponding $C_{2}=1/\sqrt{2}$ and, thus, $W_{\mu }$ is steerable when $\mu >1/\sqrt{2}\approx 0.707$. When n=3, let $B_{1}=\sigma_{x}$, $B_{2}=\sigma_{y}$, and $B_{3}=\sigma_{z}$. The corresponding $C_{3}=1/\sqrt{3}$ and, thus, $W_{\mu }$ is steerable when $\mu >1/\sqrt{3}\approx 0.577$. It can be proved that, for n=2,3, the above Bob’s observables are optimal [15,19]. When n=4, it is a little complicated, but one can let $B_{1}=\sigma_{x}$, $B_{2}=\sigma_{y}$, $B_{3}=\left(\sigma_{y}+\sqrt{3}\sigma_{z}\right)/2$, and $B_{4}=\left(\sigma_{y}-\sqrt{3}\sigma_{z}\right)/2$. The corresponding $C_{4}=\sqrt{5}/4$ and $W_{\mu }$ is steerable when $\mu >\sqrt{5}/4\approx 0.559$. In this case, the observables $\left\{B_{1},B_{2},B_{3},B_{4}\right\}$ may not be optimal. It can be concluded that the larger the number of measurement pairs, the lower bound of μ can be detected by the linear inequality. In principle, when n→∞, which can be understood as the state tomography, one can image that the critical bound for the EPR steerability can be finally found, i.e., μ>1/2 [15,19].*

This example shows the application of the linear EPR steering inequality, as well as its limitations. Firstly, the linear inequality (Equation (18)) may not give the critical bound of the EPR steerability when testing some kinds of quantum states. This makes sense as the linear inequality represents only one hyperplane in the probability space, while the sufficient and necessary condition for the EPR steerability usually requires numerous such hyperplanes. Secondly, the linear inequality (Equation (18)) closely relies on observables that would be chosen. Thus, in practice, a natural question is how to choose Alice’s and Bob’s observables such that the detection of EPR steering is efficient. Thirdly, as seen from the example, the more measurements, the better the performance of the linear inequality. However, the complexity to compute $C_{n}$ is also increasing when *n* becomes large. In fact, the method in Equation (19) to calculate $C_{n}$ needs to maximize all $a_{k}\in A_{k}$ for all *k*, which leads the complexity of $C_{n}$ exponentially increasing with *n*. Therefore, it is motivated to specify systematic techniques of choosing proper observables and obtaining $C_{n}$ more efficiently.

#### 4.1.1. Optimal Observables for Alice

Usually, Bob’s observables $\left\{B_{k}\right\}$ are fixed due to the measurement devices are trusted in his lab. Here, the problem of how Alice chooses proper measurement settings according to Bob’s observables is discussed. The main idea is that, to violate the linear inequality (Equation (18)) more obviously, Alice should choose observables such that $\langle A_{k}B_{k}\rangle$ is larger when $g_{k}>0$ and $\langle A_{k}B_{k}\rangle$ is smaller when $g_{k}<0$. In this sense, the value of $S_{n}$ can be made as large as possible so as to violate the unsteerable bound. This technique can be formulated based on the following lemma [64].

**Lemma** **3.** *For any two n×n-dimensional Hermite matrices A and B, the following equation holds,*

(27) $$\max_{U}\mathrm{tr}\left[AU^{†}BU\right]=\sum_{i=1}^{n}\alpha_{i}\beta_{i},$$

*where U is an arbitrary unitary matrix and $\alpha_{1}\geq \alpha_{2}\geq \cdots \geq \alpha_{n}$ and $\beta_{1}\geq \beta_{2}\geq \cdots \geq \beta_{n}$ are the eigenvalues of A and B, respectively.*

**Proof.**  Write $A=\sum \alpha_{i}e_{i}$ and $B=\sum \beta_{j}f_{j}$ in the diagonal form, where $\left\{e_{i}\right\}$ and $\left\{f_{j}\right\}$ are specific bases of the operator space, respectively satisfying $\mathrm{tr}\left[e_{i}e_{j}^{†}\right]=\delta_{ij}=\mathrm{tr}\left[f_{i}f_{j}^{†}\right]$ and $\sum_{i}e_{i}=I=\sum_{j}f_{j}$. Then,

(28) $$\begin{array}{cc} \max_{U}\mathrm{tr}\left[AU^{†}BU\right] & =\max_{U}\sum_{ij}\alpha_{i}\beta_{j}\mathrm{tr}\left[Ue_{i}U^{†}e_{j}\right] \end{array}$$

(29) $$\begin{array}{c} =\max_{U}\sum_{ij}\alpha_{i}\beta_{j}\mathrm{tr}\left[{\tilde{e}}_{i}e_{j}\right]=\max_{D}\sum_{ij}\alpha_{i}\beta_{j}D_{ij}. \end{array}$$

Here, $\left\{{\tilde{e}}_{i}=Ue_{i}U^{†}\right\}$ is another bases of the operator space, and it is straightforward to verify that the transition matrix $D_{ij}=\mathrm{tr}\left[{\tilde{e}}_{i}e_{j}\right]$ is a doubly stochastic matrix, i.e., $\sum_{i}D_{ij}=1$ and $\sum_{j}D_{ij}=1$. As the doubly stochastic matrix can always been written as the convex combination of permutation matrices [61], the following equation holds:

$$\max_{D}\sum_{ij}\alpha_{i}\beta_{j}D_{ij}=\max_{\sigma }\sum_{i}\alpha_{i}\beta_{\sigma \left(i\right)}=\sum_{i}\alpha_{i}\beta_{i},$$

where σ is a certain permutation. □

Then, the following technique to choose Alice’s observables $\left\{A_{k}\right\}$ can be specified [20].

**Theorem** **4.** *When the quantum state $\rho_{AB}$ is to be tested and Bob’s observables are fixed as $\left\{B_{k}\right\}$, $\langle A_{k}⊗B_{k}\rangle$ is maximal if Alice’s observables satisfy the following conditions.*

*1.*  *$A_{k}$ and ${\tilde{\rho }}_{k}={\mathrm{tr}}_{B}\left[\left(I_{A}⊗B_{k}\right)\rho_{AB}\right]$ are diagonalized in the same bases $\left\{e_{i}^{A}\right\}$.*
*2.*  *Eigenvalues of $A_{k}$ and eigenvalues of ${\tilde{\rho }}_{k}={\mathrm{tr}}_{B}\left[\left(I_{A}⊗B_{k}\right)\rho_{AB}\right]$ have the same order.*

*Then,*

$$\langle A_{k}⊗B_{k}\rangle =\sum_{i}a_{k}^{\left(i\right)}\beta_{k}^{\left(i\right)},$$

*where $\alpha_{k}^{\left(1\right)}\geq \alpha_{k}^{\left(2\right)}\geq \cdots \geq \alpha_{k}^{\left(n\right)}$ and $\beta_{k}^{\left(1\right)}\geq \beta_{k}^{\left(2\right)}\geq \cdots \geq \beta_{k}^{\left(n\right)}$ are eigenvalues of $A_{k}$ and ${\tilde{\rho }}_{k}$, respectively.*

**Proof.**  For any observables $A_{k}$ and $B_{k}$ on a quantum state $\rho_{AB}$, the expectation value of $A_{k}⊗B_{k}$ is

(30) $$\begin{array}{cc} \langle A_{k}⊗B_{k}\rangle & =\mathrm{tr}\left[A_{k}⊗B_{k}\rho_{AB}\right]={\mathrm{tr}}_{A}\left\{A_{k}{\mathrm{tr}}_{B}\left[\left(I_{A}⊗B_{k}\right)\rho_{AB}\right]\right\}=\mathrm{tr}\left[A_{k}{\tilde{\rho }}_{k}\right] \end{array}$$

(31) $$\begin{array}{c} =\mathrm{tr}\left[U_{k}D_{k}U_{k}^{†}{\tilde{\rho }}_{k}\right]=\mathrm{tr}\left[D_{k}U_{k}^{†}{\tilde{\rho }}_{k}U_{k}\right], \end{array}$$

where $U_{k}$ is a unitary matrix, $D_{k}$ is a diagonal matrix, and $A_{k}=U_{k}D_{k}U_{k}^{†}$ holds. From Lemma 3, $\mathrm{tr}\left[U_{k}D_{k}U_{k}^{†}{\tilde{\rho }}_{k}\right]$ is maximized when $U_{k}$ can diagonalize ${\tilde{\rho }}_{k}$ simultaneously, i.e., $U_{k}^{†}{\tilde{\rho }}_{k}U_{k}$ is a diagonal matrix, and $D_{k}$ has the same order of diagonal values with $U_{k}^{†}{\tilde{\rho }}_{k}U_{k}$. In this case, $\langle A_{k}⊗B_{k}\rangle =\sum_{i}a_{k}^{\left(i\right)}\beta_{k}^{\left(i\right)}$ is the maximal over all Alice’s observables, where $\alpha_{k}^{\left(1\right)}\geq \alpha_{k}^{\left(2\right)}\geq \cdots \geq \alpha_{k}^{\left(n\right)}$ and $\beta_{k}^{\left(1\right)}\geq \beta_{k}^{\left(2\right)}\geq \cdots \geq \beta_{k}^{\left(n\right)}$ are eigenvalues of $A_{k}$ and ${\tilde{\rho }}_{k},$ respectively. □

Note that, when $\rho_{k}$ contains degenerate eigenvalues, the optimal $A_{k}$ by this method are not unique. As an example, we consider the 3×3-dimensional isotropic state [23].

**Example** **2.** *The 3×3-dimensional isotropic state.*

*The 3×3-dimensional isotropic state has the following form*

(32) $$\rho_{\eta }=\eta |\phi^{+}\rangle \langle \phi^{+}|+\left(1-\eta \right)\frac{I}{9},$$

*where $|\phi^{+}\rangle =\left(|00\rangle +|11\rangle +|22\rangle \right)/\sqrt{3}$. From the partial transpose criterion [65], $\rho_{\eta }$ can be certified entangled if η>1/4. To detect its steerability, let Bob’s observables be the Gell–Mann matrices:*

(33) $$\begin{array}{lll} G_{1}=\frac{1}{\sqrt{2}}\left(\begin{array}{ccc} 0 & 1 & 0\\ 1 & 0 & 0\\ 0 & 0 & 0 \end{array}\right), & G_{2}=\frac{1}{\sqrt{2}}\left(\begin{array}{ccc} 0 & 0 & 1\\ 0 & 0 & 0\\ 1 & 0 & 0 \end{array}\right), & G_{3}=\frac{1}{\sqrt{2}}\left(\begin{array}{ccc} 0 & 0 & 0\\ 0 & 0 & 1\\ 0 & 1 & 0 \end{array}\right),\\ G_{4}=\frac{1}{\sqrt{2}}\left(\begin{array}{ccc} 0 & -i & 0\\ i & 0 & 0\\ 0 & 0 & 0 \end{array}\right), & G_{5}=\frac{1}{\sqrt{2}}\left(\begin{array}{ccc} 0 & 0 & -i\\ 0 & 0 & 0\\ i & 0 & 0 \end{array}\right), & G_{6}=\frac{1}{\sqrt{2}}\left(\begin{array}{ccc} 0 & 0 & 0\\ 0 & 0 & -i\\ 0 & i & 0 \end{array}\right),\\ G_{7}=\frac{1}{\sqrt{2}}\left(\begin{array}{ccc} 1 & 0 & 0\\ 0 & -1 & 0\\ 0 & 0 & 0 \end{array}\right), & G_{8}=\frac{1}{\sqrt{6}}\left(\begin{array}{ccc} 1 & 0 & 0\\ 0 & 1 & 0\\ 0 & 0 & -2 \end{array}\right). \end{array}$$

*Then, from Theorem 4, Alice’s observables can be chosen as $G_{k}^{A}={\left(G_{k}^{B}\right)}^{T}$, such that $\langle G_{k}^{A}⊗G_{k}^{B}\rangle =\eta /3$ obtains its maximal value and $S_{n}=\eta /3$.*

*For the LHS bound $C_{n},$ we have the following results. When n=3 and Bob chooses $G_{1}$, $G_{2}$, and $G_{3}$, the state is steerable if η>0.8660. When n=4 and Bob chooses $G_{1}$, $G_{2}$, $G_{4}$, and $G_{8}$, the state is steerable if η>0.7318. When n=5 and Bob chooses $G_{3}$, $G_{4}$, $G_{5}$, $G_{6}$, and $G_{7}$, the state is steerable if η>0.6708. When n=6 and Bob chooses $G_{1}$, $G_{2}$, $G_{3}$, $G_{4}$, $G_{5}$, and $G_{8}$, the state is steerable if η>0.6424. When n=7 and Bob chooses observables from $G_{1}$ to $G_{7}$, the state is steerable if η>0.6204. Finally, when n=8 and Bob chooses all Gell–Mann matrices, the state is steerable if η>0.5748. Note that, in this case, when Bob chooses only two observables from Gell–Mann matrices, the corresponding linear inequality will not detect any steerability of the state.*

#### 4.1.2. A Flexible Bound on Unsteerable Correlations

As discussed above, the unsteerable bound $C_{n}$ in the linear inequality from Equation (19) contains a maximization over all Alice’s measurement results. The complexity to compute $C_{n}$ is exponentially increasing with the number of *n*. This property can also be concluded from the above two examples. Therefore, when the number of measurements are large, a simpler bound is needed [66].

**Theorem** **5.** *If the result of an EPR steering test violates the following inequality*

(34) $$S_{n}=\frac{1}{n}\sum_{k=1}^{n}g_{k}\langle A_{k}B_{k}\rangle \leq C_{n}^{\prime }=\Lambda_{A}^{1/2}\Lambda_{B}^{1/2},$$

*where $g_{k}$ are some real numbers and $\Lambda_{A}\Lambda_{B}$ satisfies*

(35) $$\Lambda_{A}=\sum_{k=1}^{n}g_{k}^{2}\bar{A_{k}^{2}},\Lambda_{B}=\max_{\rho }\left\{\sum_{k=1}^{n}{\langle B_{k}\rangle }_{\rho }^{2}\right\},$$

*with $\bar{A_{k}^{2}}=\sum_{a_{k}}a_{k}^{2}p\left(a_{k}\right)$, then the correlation of the test is EPR steering. The corresponding quantum state $\rho_{AB}$ is EPR steerable, and more precisely, Alice can steer Bob.*

**Proof.**  Take in the definition of unsteerable correlation (Equation (6)),

$$P_{LHS}\left(ab|xy\right)=\int dp_{\lambda }P\left(a|x,\lambda \right)\mathrm{tr}\left[F_{b}^{y}\sigma_{\lambda }\right].$$

Then

(36) $$\begin{array}{cc} S_{n} & =\sum_{k=1}^{n}g_{k}\langle A_{k}⊗B_{k}\rangle =\int dp_{\lambda }\sum_{k=1}^{n}g_{k}\sum_{a_{k}}a_{k}P\left(a_{k}|A_{k},\lambda \right)\mathrm{tr}\left[B_{k}\sigma_{\lambda }\right] \end{array}$$

(37) $$\begin{array}{c} =\int dp_{\lambda }\sum_{k=1}^{n}g_{k}\bar{{\left(A_{k}\right)}_{\lambda }}{\langle B_{k}\rangle }_{\lambda } \end{array}$$

(38) $$\begin{array}{c} \leq \int dp_{\lambda }{\left[\sum_{k=1}^{n}g_{k}^{2}{\bar{{\left(A_{k}\right)}_{\lambda }}}^{2}\right]}^{1/2}{\left(\sum_{k=1}^{n}{\langle B_{k}\rangle }_{\sigma_{\lambda }}^{2}\right)}^{1/2} \end{array}$$

(39) $$\begin{array}{c} \leq \int dp_{\lambda }{\left[\sum_{k=1}^{n}g_{k}^{2}\bar{{\left(A_{k}^{2}\right)}_{\lambda }}\right]}^{1/2}\max_{\rho }{\left(\sum_{k=1}^{n}{\langle B_{k}\rangle }_{\rho }^{2}\right)}^{1/2} \end{array}$$

(40) $$\begin{array}{c} \leq {\left[\sum_{k=1}^{n}g_{k}^{2}\int dp_{\lambda }\bar{{\left(A_{k}^{2}\right)}_{\lambda }}\right]}^{1/2}\Lambda_{B}^{1/2} \end{array}$$

(41) $$\begin{array}{c} ={\left[\sum_{k=1}^{n}g_{k}^{2}\bar{\left(A_{k}^{2}\right)}\right]}^{1/2}\Lambda_{B}^{1/2}=\Lambda_{A}^{1/2}\Lambda_{B}^{1/2}. \end{array}$$

Here, $\bar{{\left(A_{k}\right)}_{\lambda }}=\sum a_{k}P\left(a_{k}|A_{k},\lambda \right)$ is the expectation value of $A_{k}$ under the probability distribution $P\left(a_{k}|A_{k},\lambda \right)$ and $\bar{{\left(A_{k}^{2}\right)}_{\lambda }}$ is the expectation value of $A_{k}^{2}$ under the probability distribution $P\left(a_{k}|A_{k},\lambda \right)$. The third line is based on the Cauchy–Schwarz inequality $u\cdot v\leq \left|u\right|\left|v\right|$, where we let $u=\left(\cdots g_{k}\bar{{\left(A_{k}\right)}_{\lambda }}\cdots \right)$ and v = $\left(\cdots {\left(B_{k}\right)}_{\lambda }\cdots \right)$. The fourth line comes from ${\bar{{\left(A_{k}\right)}_{\lambda }}}^{2}\leq \bar{{\left(A_{k}^{2}\right)}_{\lambda }}$ and $\sum_{k=1}^{n}{\langle B_{k}\rangle }_{\sigma_{\lambda }}^{2}\leq \max_{\rho }\sum_{k=1}^{n}{\langle B_{k}\rangle }_{\rho }^{2}$. The fifth line is due to the concavity of the function $y=x^{1/2}$. □

Compared with the bound (Equation (19)) in the linear EPR steering inequality (Equation (18)), here, the unsteerable bound $C_{n}^{\prime }$ is simpler to compute and the complexity to obtain $\Lambda_{A}$ and $\Lambda_{B}$ increases linearly with *n*. However, $C_{n}^{\prime }$ may not as tight as $C_{n}$, i.e., some steerable states may be detectable by bound $C_{n}$ but not with bound $C_{n}^{\prime }$.

### 4.2. EPR Steering Inequality Based on Local Uncertainty Relations

For a random variable $X=\left\{x_{i}\right\}$, the variance is defined as $\delta^{2}\left(X\right)=\bar{X^{2}}-{\bar{X}}^{2}$, where $\bar{X^{2}}=\sum_{i}p\left(x_{i}\right)x_{i}^{2}$ is the mean of the square of *X* and ${\bar{X}}^{2}={\left(\sum_{i}p\left(x_{i}\right)x_{i}\right)}^{2}$ is the square of the mean of *X*. For any random variable *X*, $\delta^{2}\left(X\right)\geq 0$ always holds. In quantum mechanics, the variance describes the uncertainty of measurement results. For instance, consider the projective measurement $M=\sum_{k}m_{k}\Pi_{k}$, where $\Pi_{k}$ are projectors and $m_{k}$ are the corresponding outcome. The variance of measurement results $\left\{m_{i}\right\}$ on a quantum state ρ is in the form of $\delta^{2}{\left(M\right)}_{\rho }={\langle M^{2}\rangle }_{\rho }-{\langle M\rangle }_{\rho }^{2}$, where ${\langle M\rangle }_{\rho }=\mathrm{tr}\left[M\rho \right]$ is the expectation value of measurement *M* on ρ and ${\langle M^{2}\rangle }_{\rho }=\mathrm{tr}\left[M^{2}\rho \right]$ is the expectation value of the square of measurement *M* on ρ. In the following, the subscript ρ is omitted for simplicity. The uncertainty relation can be described as, for a set of measurements $\left\{M_{i}|i=1,\cdots ,n\right\}$, the sum of variances is larger than a certain value, i.e., $\sum_{i}\delta^{2}\left(M_{i}\right)\geq C_{M}$ with $C_{M}=\min_{\rho }\sum_{i}\delta^{2}{\left(M_{i}\right)}_{\rho }$. In a nontrivial case, where $\left\{M_{i}\right\}$ has no common eigenvectors, $C_{M}$ is positive, i.e., $C_{M}>0$ [67,68,69].

In the EPR steering test, only Bob’s measurements are assumed to be quantum. Then, the local uncertainty relations (LUR) on Bob’s side can help to certify EPR steering correlation [23].

**Theorem** **6** (Steering inequality based on LUR). *If the result of an EPR steering test violates the following inequality*

(42) $$\sum_{k=1}^{n}\delta^{2}\left(\alpha_{i}A_{i}+B_{i}\right)\geq C_{B},$$

*where $\alpha_{i}$ are some real numbers and $C_{B}=\min_{\rho }\sum_{i}\delta^{2}{\left(B_{i}\right)}_{\rho }$, then the correlation of the test is EPR steering. The corresponding quantum state $\rho_{AB}$ is EPR steerable, and more precisely, Alice can steer Bob.*

**Proof.**  Generally, for any two random variables *X* and *Y*, let $p\left(xy\right)$ be the joint probability distribution and $p\left(y|x\right)=p\left(xy\right)/p\left(x\right)$ be the conditioned probability distribution. Then, the variance of *Y* satisfies

(43) $$\begin{array}{cc} \delta^{2}\left(Y\right) & =\sum_{y}p\left(y\right)y^{2}-{\left[\sum_{y}p\left(y\right)y\right]}^{2} \end{array}$$

(44) $$\begin{array}{c} =\sum_{y,x}p\left(x\right)p\left(y|x\right)y^{2}-{\left[\sum_{y,x}p\left(x\right)p\left(y|x\right)y\right]}^{2} \end{array}$$

(45) $$\begin{array}{c} \geq \sum_{x}p\left(x\right)\left[\sum_{y}p\left(y|x\right)y^{2}-{\left(\sum_{y}p\left(y|x\right)y\right)}^{2}\right] \end{array}$$

(46) $$\begin{array}{c} =\sum_{x}p\left(x\right)\delta^{2}{\left(y\right)}_{x}, \end{array}$$

where the third line comes from the concavity of function $f\left(t\right)=t^{2}$ and $\delta^{2}{\left(y\right)}_{x}$ is the variance of *Y* under the distribution $\left\{p\left(y|x\right)\right\}$. Now, consider the definition of unsteerable correlation

$$P_{LHS}\left(ab|xy\right)=\int d\lambda p_{\lambda }P\left(a|x,\lambda \right)\mathrm{tr}\left[F_{b}^{y}\sigma_{\lambda }\right].$$

One has

(47) $$\begin{array}{cc} \sum_{i}\delta^{2}\left(\alpha_{i}A_{i}+B_{i}\right) & \geq \sum_{i}\int d\lambda p_{\lambda }\delta^{2}{\left(\alpha_{i}A_{i}+B_{i}\right)}_{\lambda } \end{array}$$

(48) $$\begin{array}{c} =\sum_{i}\int d\lambda p_{\lambda }\left[\bar{{\left(\alpha_{i}A_{i}+B_{i}\right)}_{\lambda }^{2}}-{\left(\alpha_{i}{\bar{A}}_{i}+{\bar{B}}_{i}\right)}_{\lambda }^{2}\right] \end{array}$$

(49) $$\begin{array}{c} =\sum_{i}\int d\lambda p_{\lambda }{\left[\alpha_{i}^{2}\left(\bar{A_{i}^{2}}-{\bar{A}}_{i}^{2}\right)+\left(\bar{B_{i}^{2}}-{\bar{B}}_{i}^{2}\right)\right]}_{\lambda } \end{array}$$

(50) $$\begin{array}{c} =\sum_{i}\int d\lambda p_{\lambda }\left[\alpha_{i}^{2}\delta^{2}{\left(A_{i}\right)}_{\lambda }+\delta^{2}{\left(B_{i}\right)}_{\sigma_{\lambda }}\right] \end{array}$$

(51) $$\begin{array}{c} \geq \sum_{i}\int d\lambda p_{\lambda }\left[0+\sum_{i}\delta^{2}{\left(B_{i}\right)}_{\sigma_{\lambda }}\right]\geq C_{B}, \end{array}$$

where trivial results $\delta^{2}{\left(A_{i}\right)}_{\lambda }⩾0$ is used. □

Here, $\left\{\alpha_{i}\right\}$ are some flexible real variables. For a certain probability distribution $\left\{P\left(a_{k}b_{k}|A_{k}B_{k}\right)\right\}$ generated from an EPR steering test, the optimal $\left\{\alpha_{i}\right\}$ can be calculated such that the inequality from Equation (42) is maximally violated. For each term in Equation (42), $\delta^{2}\left(\alpha_{i}A_{i}+B_{i}\right)=\alpha_{i}^{2}\delta^{2}\left(A_{i}\right)+2\alpha_{i}C\left(A_{i},B_{i}\right)+\delta^{2}\left(B_{i}\right)$ holds where $C\left(A_{i},B_{i}\right)=\langle A_{i}B_{i}\rangle -\langle A_{i}\rangle \langle B_{i}\rangle$ is the covariance. Therefore, $\delta^{2}\left(\alpha_{i}A_{i}+B_{i}\right)$ can be seen as a quadratic polynomial of $\alpha_{i}$, from which the optimal $\alpha_{i}$ can be obtained, i.e.,

$$\alpha_{i}=\left\{\begin{array}{cc} -C\left(A_{i},B_{i}\right)/\delta^{2}\left(A_{i}\right), & \mathrm{if}\;\delta^{2}\left(A_{i}\right)\neq 0;\\ -\delta^{2}\left(B_{i}\right)/2C\left(A_{i},B_{i}\right), & \mathrm{if}\;\delta^{2}\left(A_{i}\right)=0,C\left(A_{i},B_{i}\right)\neq 0;\\ 0, & \mathrm{if}\;\delta^{2}\left(A_{i}\right)\neq 0,C\left(A_{i},B_{i}\right)=0. \end{array}\right.$$

It is noteworthy that, here, like the case in the linear inequality of Equation (34), the complexity to compute unsteerable bound $C_{B}$ also increases linearly with the number of measurements *n*, better than the case in inequality (Equation (18)), where the complexity increases exponentially with *n*.

**Remark** **2.** *The use of LUR in quantum correlations.*

*In the case of EPR steering, the inequality from Equation (42) shows that, for unsteerable correlations, the uncertainty of the total system AB is always larger than that of one subsystem B. This conclusion is consistent with the definition of LHS models, where only Bob has the quantum description. One property of EPR steering is, thus, that the uncertainty of the correlated measurement results can be less than the uncertainty of one subsystem. In this sense, the violation of LUR indicates the amount of quantum correlations.*

*Furthermore, if quantum entanglement is considered in this fashion, for any separable states $\sigma_{AB}^{SEP}=\sum_{k}p_{k}\sigma_{k}^{A}⊗\sigma_{k}^{B}$, it has been proved that*

(52) $$\sum_{i}\delta^{2}{\left(A_{i}+B_{i}\right)}_{SEP}\geq C_{A}+C_{B},$$

*where $C_{A}=\min_{\rho }\sum_{i}\delta^{2}{\left(A_{i}\right)}_{\rho }$ [70]. That is, in the case of quantum separable states, where both Alice and Bob can be described as quantum but classically correlated, the uncertainty of the total system is always larger than the sum of the local uncertainty relations of all subsystems.*

*However, for the nonlocality, the probability distribution of LHV models always satisfies*

(53) $$\sum_{i}\delta^{2}\left(A_{i}+B_{i}\right)\geq 0,$$

*which is a trivial result, and no violation can be detected. In fact, formulating a nonlinear form of Bell inequalities is a difficult problem.*

*It is noteworthy that in Reference [43], the violation of the CHSH inequality [62] can be restricted by the so-called fine-grained uncertainty relations combined by a properly-defined steerability. Such a restriction holds only when a specific form of the Bell inequalities are selected [44]. Different from the variance-based uncertainties discussed here or entropies [24], the fine-grained uncertainty relation are described in a linear form of the set of measurement observables, which can also be used as the certification of EPR steering [25].*

**Example** **3.** *Bell diagonal states*

*Bell diagonal states has the following simple form,*

(54) $$\rho_{c}=\frac{1}{4}\left[I+\sum_{j}c_{j}\sigma_{j}⊗\sigma_{j}\right],$$

*where $\left\{\sigma_{j},j=x,y,z\right\}$ is the set of Pauli matrices. In another form, $\rho_{c}$ can be written in the diagonal form*

(55) $$\rho_{c}=t_{1}|\psi^{+}\rangle \langle \psi^{+}|+t_{2}|\psi^{-}\rangle \langle \psi^{-}|+t_{3}|\phi^{+}\rangle \langle \phi^{+}|+t_{4}|\phi^{-}\rangle \langle \phi^{-}|,$$

*where $|\psi^{\pm }\rangle =\left(|00\rangle \pm |11\rangle \right)/\sqrt{2}$ and $|\phi^{\pm }\rangle =\left(|01\rangle \pm |10\rangle \right)/\sqrt{2}$ are four Bell states and $\sum_{i}t_{i}=1$.*

*If three Pauli matrices are selected as the observables, the linear EPR steering inequality (18) can be simplified as $\left|\sum_{i}\omega_{i}\langle \sigma_{i}^{A}⊗\sigma_{i}^{B}\rangle \right|<\sqrt{3}$ with $\omega_{i}\in \left\{\pm 1\right\}$. Here, the absolute value and binary $\omega_{i}$ suggest that there are a set of linear inequalities. The violation implies that $\rho_{c}$ is steerable if $\left|c_{x}\pm c_{y}\pm c_{z}\right|>\sqrt{3}$. Nevertheless, the EPR steering inequalities (42) based on LUR can be optimized as $\sum_{i}\delta^{2}\left(\sigma_{i}^{B}\right)-C^{2}\left(\sigma_{i}^{A},\sigma_{i}^{B}\right)/\delta^{2}\left(\sigma_{i}^{A}\right)⩾2$, the violation of which implies $\sum_{i}c_{i}^{2}>1$. As a comparison, it can be verified that, in this example, the inequality based on LUR certifies a larger steerable region of Bell diagonal states than the linear inequality [23].*

### 4.3. Realignment Method

From the EPR steering inequality based on LUR, the realignment method for certifying entanglement also works for the EPR steering case. Generally, the realignment criterion [71] or the computable cross-norm criterion [72] are important techniques to certify bound quantum entanglement, i.e., entangled states with a positive partial transpose. Mathematically, the realignment is a map on a quantum state $\rho_{AB}$ such that $R\left(\rho_{AB}\right):\rho_{AB}\mapsto \langle m|\langle \mu |R\left(\rho_{AB}\right)|n\rangle |v\rangle =\langle m|\langle n|\rho_{AB}|v\rangle |\mu \rangle$. If $\rho_{AB}$ is separable, then the trace norm of the matrix $R\left(\rho \right)$ is not larger than 1.

To obtain the norm of $R\left(\rho \right)$, one can seek for the complete set of local orthogonal observables (LOOs). A complete set of LOOs is a collection of observables $\left\{G_{k}\right\}$ satisfying $G_{k}^{†}=G_{k}$, $\mathrm{tr}\left[G_{k}G_{l}\right]=\delta_{kl}\;$, and $\sum_{k}G_{k}^{2}=I$. Indeed, $\left\{G_{k}\right\}$ forms a complete set of orthonormal bases for the corresponding operator space. Then, a state ρ can be written as $\rho =\sum_{k}\mu_{k}G_{k}$, where $\mu_{k}=\mathrm{tr}\left[\rho G_{k}\right]$. For example, in the case of qubits, the identity matrix and three Pauli matrices form a complete set of LOOs, and in the case of qutrits, the identity matrix and eight Gell-Mann matrices form a complete set of LOOs.

For any bipartite quantum state $\rho_{AB}$, suppose that the maximal dimension of Alice’s Hilbert space and Bob’s Hilbert space is *d*. Let the complete sets of LOOs for Alice’s operator space and Bob’s operator space be $\left\{{\tilde{G}}_{k}^{A}\right\}$ and $\left\{{\tilde{G}}_{k}^{B}\right\}$, respectively. Then, $\rho_{AB}$ can always be written as $\rho_{AB}=\sum_{kl}\mu_{kl}{\tilde{G}}_{k}^{A}⊗{\tilde{G}}_{k}^{B}$, where $\mu_{kl}=\mathrm{tr}\left[\rho_{AB}{\tilde{G}}_{k}^{A}⊗{\tilde{G}}_{k}^{B}\right]$. The singular value decomposition on the matrix $\mu =\left(\mu_{kl}\right)$ yields $\mu =S\lambda T^{T}$, where $\lambda =\mathrm{diag}\left\{\lambda_{1},\cdots ,\lambda_{d^{2}}\right\}$ is the diagonal matrix with $\lambda_{k}\geq 0$, $S=\left(s_{ij}\right)$ and $T=\left(t_{ij}\right)$ are two orthogonal matrices, i.e., $SS^{T}=TT^{T}=I$. Take $\mu =S\lambda T^{T}$ into the expression of $\rho_{AB}$, and finally, the Hilbert–Schmidt decomposition of $\rho_{AB}$ can be obtained:

(56) $$\rho_{AB}=\sum_{k}\lambda_{k}G_{k}^{A}⊗G_{k}^{B},$$

where $G_{k}^{A}=\sum_{m}s_{mk}{\tilde{G}}_{m}^{A}$ and $G_{k}^{B}=\sum_{m}t_{mk}{\tilde{G}}_{m}^{B}$. It can be verified that $\left\{G_{k}^{A}\right\}$ and $\left\{G_{k}^{B}\right\}$ are another two complete sets of LOOs, and $\lambda_{k}=\mathrm{tr}\left[\rho_{AB}G_{k}^{A}⊗G_{k}^{B}\right]$.

In a certifying entanglement, if $\rho_{AB}$ is separable, then the realignment [71,72] method guarantees that

(57) $$\sum_{k}\lambda_{k}⩽1.$$

In certifying EPR steering, a similar result can be concluded [23].

**Theorem** **7** (Realignment for EPR steering). *If $\rho_{AB}=\sum_{k}\lambda_{k}G_{k}^{A}⊗G_{k}^{B}$ satisfies*

(58) $$\sum_{k}\lambda_{k}>\sqrt{d},$$

*then $\rho_{AB}$ is EPR steerable. In this case, Alice can steer Bob and Bob can also steer Alice.*

**Proof.**  From the EPR steering inequality based on LUR, for a bipartite quantum state $\rho_{AB}=\sum_{k}\lambda_{k}G_{k}^{A}⊗G_{k}^{B}$, let Alice’s and Bob’s observables be $\left\{G_{k}^{A}\right\}$ and $\left\{G_{k}^{B}\right\}$ and $g_{k}=-g$. The violation of Equation (42) implies $g^{2}d+d-2g\sum_{k}\lambda_{k}-\sum_{k}{\left(g\langle G_{k}^{A}\rangle -\langle G_{k}^{B}\rangle \right)}^{2}<d-1$. A sufficient condition of this inequality is omitting the quadratic term, i.e., $g^{2}d+d-2g\sum_{k}\lambda_{k}<d-1$. Finally, let $g=\sum_{k}\lambda_{k}/d$, and the inequality (58) is concluded. □

Different from the linear inequality and the inequality based on LUR, the realignment method does not require an EPR steering test. For any quantum state $\rho_{AB}$, there is a possibility that one can know whether this state is EPR steerable or not, regardless of how to certify it in the test. A limitation is that, as a corollary of the inequality, the realignment method will not perform better than the inequality.

In the entanglement case, where the state is entangled if the value $\sum_{k}\lambda_{k}$ is larger than 1. Here, this quantity should be larger than $\sqrt{d}$ to certify the EPR steerability. Although the realignment method can certify positive partial transpose (PPT) entanglement, it remains an open question if it can certify PPT EPR steering, i.e., EPR steerable states with PPT. Note that there have been numerical results proving the existence of such states [73,74].

## 5. Summary

In this survey, the basic technique to discuss and certify EPR steering is discussed. Particularly, the box framework and trust-untrust scenario is adopted. The linear criterion and local-uncertainty-relation-based criterion are summarized. Both criteria are constructed in an experimentally friendly manner, i.e., they can be directly applied in real experiments for arbitrary measurement settings and arbitrary outcomes, with a reduced complexity to obtain the unsteerable bound. Moreover, an analytical method for the optimization of EPR steering detection is also maintained. Furthermore, from these criteria, LUR are shown to play an important role in the correlation exhibition of quantum bipartite systems.

There have also been other useful criteria, as has been listed in Section 1. Most of them are formulated in the same fashion as introduced in this survey. Therefore, the discussed techniques to find a computable unsteerable bound and optimal observables can be directly applied. There still remains an open problem of how much entanglement is sufficient for EPR steering and how much EPR steering is sufficient for nonlocality. Solving this problem would technically advance the realization of nonlocality-based quantum protocols and finally contributes to the application of quantum information technologies.

## Abbreviations

The following abbreviations are used in this manuscript:

EPR	Einstein–Podolsky–Rosen
LHV	local hidden variable
LHS	local hidden state
LOO	local orthogonal observable
LUR	local uncertainty relations
POVM	positive operator valued measure
PPT	positive partial transpose
